# Integrated omics reveal the mechanisms underlying softening and aroma changes in pear during postharvest storage and the role of melatonin

**DOI:** 10.1186/s12870-025-06714-4

**Published:** 2025-05-22

**Authors:** Jiayu Xu, Ying Zhang, Hongliang Huo, Dan Qi, Xingguang Dong, Luming Tian, Chao Liu, Yufen Cao

**Affiliations:** Research Institute of Pomology, Chinese Academy of Agricultural Sciences, Xingcheng, Liaoning 125100 China

**Keywords:** Zaoshu Shanli, Whole transcriptome, Firmness, Transmission electron microscopy, Ethylene, Metabolite, CeRNA

## Abstract

**Background:**

*Pyrus ussuriensis* Maxim. are rich in nutrients, with a pleasant aroma and postharvest softening properties. Postharvest softening influences shelf life of fruit and fruit quality. Melatonin is a natural and safe preservative, which can effectively maintain fruit quality after harvesting, and delay softening of fruit. The aim of study was to elucidate mechanism of pear fruit softening and fruit aroma during postharvest storage and effect of melatonin.

**Results:**

Ethylene production rate, respiration rate, weight loss of fruit, soluble solid content, titratable acidity were assessed, and transmission electron microscopy, metabolite profiling, and whole-transcriptome RNA-sequencing were performed. Four important pathways that pentose and glucuronate interconversion, galactose metabolism, sphingolipid metabolism and the starch and sucrose metabolism pathway were involved in pear fruit softening. Ethylene production pathway-related genes, such as *ACS* and *ACO* were involved in pear fruit softening and expression of that under exogenous melatonin treatment were slightly inhibited. Fruit aroma changed after storage mainly through lipoxygenase pathway under ddH_2_O treatment and exogenous melatonin treatment changed composition of volatile organic compounds. CeRNA networks associated with pear softening and aroma were established. Mdm-miR159a, mdm-miR396a/b-p3 and mdm-miR408a were found to modulate both fruit softening and aroma formation through ceRNA analysis. Mdm-miR10988-p3 was functionally diverse and as major regulatory components in ceRNA network.

**Conclusions:**

This study indicated that degradation of cell wall caused pear fruit softening, lipoxygenase pathway mainly affected change of fruit aroma during postharvest storage and exogenous melatonin treatment could improve fruit firmness after storage and alter pear's aroma. The mechanism underlying these effects was elucidated, providing theoretical basis for study of pear fruit softening and preservation technology.

**Supplementary Information:**

The online version contains supplementary material available at 10.1186/s12870-025-06714-4.

## Introduction

The pear (*Pyrus*) is one of the most valuable genera of fruit trees found in temperate regions. It has multiple surviving cultivars and wild species [1]. Pears are rich in antioxidants, vitamins, minerals, nutrients, and various phytochemicals, which have significant medicinal properties [2]. Pears are classified as climacteric fruit with high post-harvest ethylene production levels [3] and respiration rates [4] that cannot be effectively suppressed. Most fleshy fruits have a characteristic called softening, and the actual ripening process is very complex, with various intertwining biochemical, physiological, and organoleptic changes that alter fruit coloration, firmness, texture, aroma, flavor, and nutritional characteristics [5–7].

After fruit storage, reduced firmness and changes in flavor-related compounds are observed [8, 9]. Reduced firmness is a salient change that promotes fruit’s sensitivity to mechanical damage, which shortens their shelf lives [10]. Furthermore, depending on the species and desirable cultivar, some degree of softening is acceptable; however, when excessive softening often results in postharvest decay and consumer rejection. Fruit softening is caused by changes in cell wall, degradation of the cell wall composition and structure have been ascribed to the activity of cell wall degrading enzymes like *PME* and cellulase on the polysaccharide content such as pectin, hemicelluloses and cellulose in the cell wall [11]. The peak production of ethylene is accompanied by volatiles burst, pulp softening by cell wall decomposition and finally lead to a increase in softening and reduction in storage life of fruit [12]. *PME* had a potential role in the fruit softening in *Pyrus communis* L. [13]. *SlPG49* overexpression promotes tomato fruit softening, while *FaPG1* silencing in strawberry has the opposite effect by inhibiting cell wall degradation, improving fruit firmness, and extending the shelf life of fruit [14, 15]. Meanwhile, European pears softening is related to *PG* gene expression [16]. *β-GAL* have been shown to be correlated with pear fruit softening, and the divergent expressions of *β-GALs* exhibit characteristics of differential regulation during fruit softening in various pear cultivars [17, 18].

Exogenous melatonin application can delay the softening of many fleshy fruits and impact their quality during storage, which has been suggested to regulate the different underlying mechanisms of fruit maturity [19]. Melatonin was observed to inhibit the rate of ethylene production in *Pyrus communis* L. fruit during postharvest storage [20] and decreased rate of polygalacturonase and cellulase upregulation during storage at room temperature in pear fruits [21]. Exogenous melatonin treatment was found to alter abundance of some volatile organic compounds in pear during storage [22] and also delayed softening and reduce peak value of endogenous ethylene release rate in ‘Xinli No.7’pear fruit during room temperature storage [23]. During apple softening, melatonin can reduce ethylene production by downregulating genes related to ethylene biosynthesis, thereby delaying fruit softening [24].

Several studies have indicated that noncoding RNAs (ncRNAs) was involved in varieties of biological processes. Chen et al. (2021) reported the importance of lncRNAs on control of kiwi fruit softening [25]. CircRNA174 has been found to regulate the biosynthesis of aroma in tea [26]. A study reported that the ripening of peppers was associated with changes in the expression of 43 miRNAs, 125 circRNAs, and 366 lncRNAs [27]. Lakhwani et al. (2020) showed miRNAs regulated fruit softening and aroma biosynthesis during banana ripening [28].

Overall, postharvest softening of pear fruit was a very important biological phenomenon and exogenous melatonin treatment can influence the degree of fruit softening and modulate aroma under certain conditions and studies rarely involved ncRNAs. This study investigated the physiological and molecular mechanism underlying with and without melatonin application effects on fruit softening and aroma by assessing ethylene production, mRNA, ncRNA (lncRNA, miRNA, and circRNA) levels, TEM analysis, metabolomics studies, and ceRNA network analysis. This study provides a theoretical basis for comprehensive research on fruit softening and aroma after storage.

## Materials and methods

### Fruit material and treatment

ZSSL pears were provided by the National Germplasm Repository of Pear and Apple (Xingcheng, China). It was selected as an experimental material because of low flesh firmness after storage and better fruit quality in wild *Pyrus ussuriensis* Maxim. Fruits, which were uniformly sized, had a good appearance and were disease-free at harvest, were selected from different trees and immediately transported to the laboratory. A total of 210 fruits were selected and assigned to two groups [105 fruits per lot, triplicate (35 fruits per replicate)]. The two groups were immersed in ddH_2_O and 0.5 mmol L^−1^ melatonin in the dark to prevent melatonin degradation for 2 h, respectively. Then, the fruits were dried and stored for 10 d at room temperature (25 ± 1 °C). Subsequently, 12 random fruits from each group were analyzed at 0, 2, 4, 6, 8, and 10 d after melatonin treatment to assess ethylene and respiration rates. Furthermore, firmness, weight per fruit, TA, and SSC were measured in the two groups at 0 and 10 d. From each group, 3 replicates and 3 fruits per replicate were assessed. Moreover, from the two groups, the fruit flesh tissue was collected at 0 d and 10 d, frozen in liquid nitrogen, and immediately stored at -80 °C for further analysis, from each group, 3 replicates and 3 fruits per replicate were assessed.

### Analyses of fruit firmness, SSC, TA, and weight per fruit

After storage as required, the firmness of the fruit flesh was measured in newtons (N) using a universal firmness testing machine (test probe: 8 mm; GY-4, HANDPI, China). Fruit weight was measured using an electronic balance (AL204-IC, METTLER TOLEDO, Shanghai) after being stored for an optimized time. Soluble solids content (SSC) and titratable acidity (TA) were determined using pear fruit juice. For SSC determination, approximately 0.8 mL of freshly extracted juice was placed in the sample well of refractometer (PAL-1 (ATAGO, Japan)), then data were recorded after pressing start button. TA quantification was performed using a Metrohm 905 Titrando system with 0.1 mol L^−1^ NaOH titrant. Specifically, 3.0 g of homogenized juice was diluted with 50 mL ultrapure water and titrated potentiometrically to pH 8.2. The NaOH titrant was standardized against certified potassium hydrogen phthalate (KHP) as primary standard.

### Ethylene production and respiration rate analyses

The rate of ethylene production (μL kg^−1^ h^−1^) and the respiration (mg kg^−1^ h^−1^) in the fruits were assessed via a SP-7890 gas chromatograph (Shandong Lunan Ruihong Instrument Co., Ltd). High-purity N_2_ was employed as the carrier gas in these analyses, with a flow rate of 55—58 mL min^−1^. H_2_ and air, at 0.2 MPa and 0.1 MPa, respectively. The conversion over was maintained at 360℃, and the temperature of the packed stainless steel column was 120℃. Detection was performed at 140℃ with an FID detector. FID detector in series with nickel-catalyst reformer for carbon dioxide. Briefly, fruit from different treatment conditions were placed in sealed plastic boxes of identical volume for 1 h at room temperature. Then, a syringe was used to collect 1 mL of gas for analysis. Three replicates were evaluated for each treatment condition, and the average measurement was reported.

### Transmission electron microscopy (TEM)

Pear flesh samples from the 0 d, ddH_2_O, and melatonin (0.5 mM) groups were fixed in 5% glutaraldehyde and 4% paraformaldehyde, rinsed using post-PB buffer, treated with acetone for dehydration, embedding in resin, polymerized, sectioned, subjected to dual-staining using dioxygen acetate and lead catalase, and then imaged using an HT-7700 TEM (Hitachi, Japan) for ultrastructural analysis.

### Volatine organic compound extraction

To extract organic volatile compounds, powdered samples (500 mg, 1 mL) were transferred into a 20 mL head-space vial (Agilent, CA, USA) containing a NaCl-saturated solution to block enzymatic processes. The vials were then sealed using crimp-top caps with TFE-silicone headspace septa from Agilent, incubated at 60 °C for 5 min, and treated with a 120 µm DVB/CWR/PDMS fiber from Agilent for 15 min at 60 °C. Three replicates were analyzed for each sample.

### GC–MS analyses

Volatile organic compound desorption from fibers was carried out via the injection port of the GC instrument (Model 8890; Agilent) for 5 min at 250 °C in splitless mode. The compounds were identified and quantified via the Agilent Model 8890 GC and a 7000D mass spectrometer (Agilent) fitted with a 30 m × 0.25 mm × 0.25 μm DB-5MS (5% phenyl-polymethylsiloxane) capillary column. Helium served as the carrier gas at a linear speed of 1.2 mL min^−1^. The oven temperature rose from 40 °C (3.5 min), at 10 °C/min to 100 °C, at 7 °C min^−1^ to 180 °C, at 25 °C min^−1^ to 280 °C, and was held at this level for 5 min. The injector was kept at 250 °C during this time. The mass spectra were recorded at 70 eV using electron impact ionization mode, with the quadrupole mass detector, ion source, and transfer line temperatures set at 150 °C, 230 °C, and 280 °C, respectively. Analyte detection and quantification were performed using the selected ion monitoring (SIM) mode. The analyses were performed by Maiwei Metabolic Biotechnology Co., Ltd. (Wuhan, China).

### Differentially metabolites selection

The differential metabolites between the groups were identified based on a VIP > 1 and a |Log2FC|≥ 1.0. The VIP values were derived from the OPLS-DA plots produced using the MetaboAnalystR program, which included encompassed score plots and permutation plots. Before the OPLS-DA analysis, data were subjected to mean centering and log transformation. To prevent overfitting, 200 permutation tests were employed.

### RNA extraction, sequencing, and library preparation

TRIzol (Thermo Fisher) was used to isolate total RNA from the ZSSL pear’s flash samples acquired from the 0 d, ddH_2_O, and melatonin (0.5 mM) groups. After rRNA removal, the remaining RNA primarily consisted of mRNAs and ncRNAs. Then, U-labeled second-stranded DNAs were treated with a heat-labile UDG enzyme (NEB, cat.m0280, USA), and ligated products were amplified by PCR. For cDNA library sequencing, an Illumina NovaSeq 6000 instrument was used. A total of 3 biological replicates were assessed per sample. RNA sequencing (RNA-seq) was carried out by creating 9 libraries (for 3 groups with 3 replicates). Sequencing was carried out by Maiwei Metabolic Biotechnology Co., Ltd. (Wuhan, China).

### Transcriptomic analyses

For quality analyses of control raw sequencing data, FastQC (http://www.bioinformatics.babraham.ac.uk/projects/fastqc/,v.0.11.9) was employed. Furthermore, the clean reads were mapped to the previously published *Pyrus communis* genome (https://www.rosaceae.org/rosaceae_downloads/Pyrus_communis/Pcommunis_DH_genome.v2.0/) via HISAT2 ( https://daehwankimlab.github.io/hisat2/,hisat2-2.2.1). Then, StringTie (http://ccb.jhu.edu/software/stringtie/,v.stringtie-2.1.6) was employed to further map the read assembly.

### Differentially expressed mRNA (DEmRNA) identification and functional enrichment analysis

FPKM values were used to assess mRNA expression levels at a significance threshold of q < 0.05 using the DESeq2R package (v 1.22.2). The GO annotation and KEGG pathway analyses were performed on the DEmRNAs using the omicstudio platform (https://www.omicstudio.cn/tool).

### Identification of miRNA, lncRNA, and circRNA, as well as target gene prediction

Clean short RNA readings after removing low-quality, adaptors (< 18 nt), and poly(A) sequences were assessed to identify miRNAs. The non-miRNA reads were removed from raw reads using the Rfam (v12.0, ftp://ftp.ebi.ac.uk/pub/databases/Rfam) and Repbase (v22.07, http://www.girinst.org/repbase) databases. The miRbase (v.22.1, http://www.mirbase.org/) was employed to assess known microRNAs (miRNAs) using the remaining clean reads. The distinctive hairpin structure linked to pre-miRNAs was utilized to forecast novel miRNAs using the RNAfold program (http://rna.tbi.univie.ac.at/CGI-bin/RNAfold.CGI). Differentially expressed miRNAs (DEmiRNAs) were found by computing normalized miRNA expression values (Student's t-test, *p* < 0.05). Target genes linked to these DEmiRNAs were predicted using Gstar (v1.0).

For lncRNA identification, the overlap between CNCI2.0 (https://github.com/www-bioinfo-org/CNCI#install-cnci) and the Coding Potential Calculator (CPC0.9-r2; http://cpc2.cbi.pku.edu.cn) was considered novel potential lncRNAs, which were detected based on ≥ 200 bp long transcripts with ≥ 1 exon number. FPKM values were employed to evaluate the expression of these lncRNAs, and DESeq2 (v 1.22.2) was utilized to identify the differentially expressed lncRNAs (DElncRNAs). Then, the possible cis- and trans-regulatory target mRNAs associated with these DElncRNAs were predicted by assessing chromosomal positioning. Trans-target genes were evaluated based on correlations between lncRNA and the expression levels of the protein-coding genes, whereas cis-target genes were defined as any protein-coding genes within a 10 kb distance upstream or downstream of a specific lncRNA [29, 30].

For circRNA identification, Bowtie2 was used to remove mapped rRNA reads, and all remaining unmapped reads were retained for analysis. The anchor reads were produced by gathering both ends of each unmapped read (20 bp by default). Then, circRNAs were identified using CIRCExplorer2 (2.2.6, default) and CIRI (2.0.2, default) by aligning the anchor read to the reference genome [31]. The EdgeR software (v3.22.5) was used to identify differentially expressed circRNAs (DEcircRNAs). The levels of circRNA expression were determined using the number of circular reads/number of mapped reads (in billions)/read length approach.

### Construction of mRNA, ceRNA, and metabolite regulation networks

Pearson correlation coefficient (PCC) values were used to analyze the DCC and DEG relationships, with the following significance criteria: |PCC|> 0.8, *p* < *0.05*. Gene-metabolite networks were shown using Cytoscape (v 3.7.1) [32]. Furthermore, Targetfinder and Ssearch36 (36.3.6) were utilized to predict miRNA-lncRNA, miRNA-mRNA, and miRNA-circRNA pairs. Moreover, Cytoscape was used to visualize interaction network maps.

### Statistical analysis

For all the statistical analyses, GraphPad Prism 5 was employed and the data represented an average of three replicates.

## Results

### Analyses of pear fruit firmness, SSC, TA, weight, ethylene production, respiration, and ultrastructural features

**Fig. 1:**
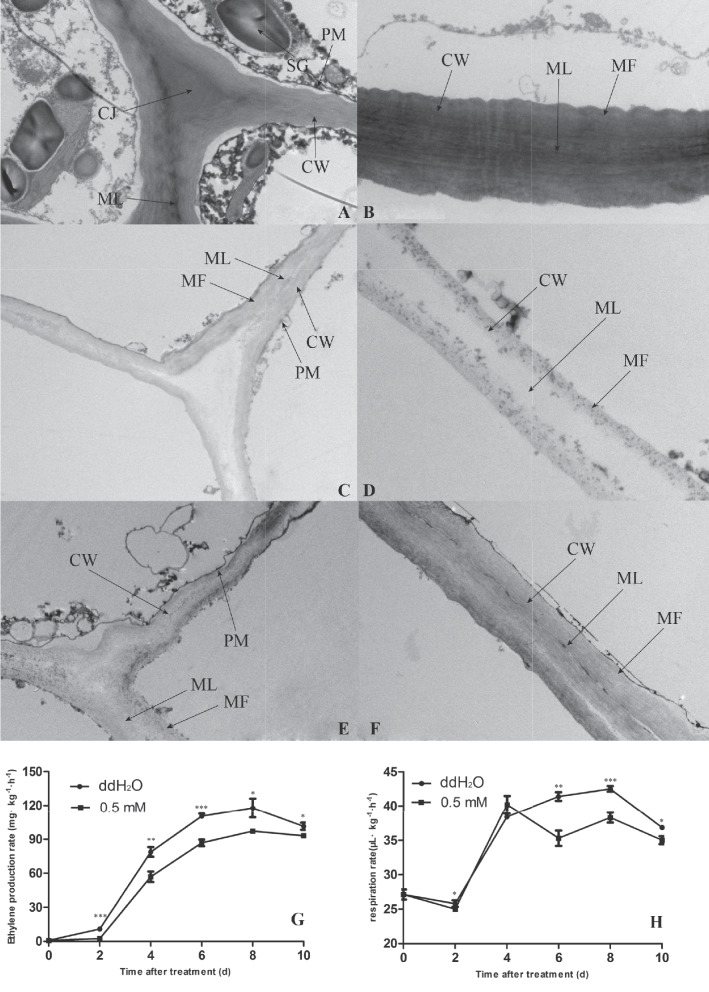
TEM analyses (**A**-**D**, **E**–**F**), Ethylene production rate (**G**) and respiration rate (**H**) of pear fruit from the ddH_2_O and exogenous melatonin treatment. **A**, **B** 0 d, (**C**, **D**) ddH_2_O, and (**E**, **F**) melatonin (0.5 mM) groups. Asterisks represent values that are significantly different (*P < 0.05, **P < 0.01 and ***P < 0.001) between two treatments

Fruit flesh firmness in 0.5 mM melatonin and ddH_2_O was reduced by 51% and 78%, respectively, indicating melatonin treatment slowed down fruit softening after storage, while weight per fruit and contents of SSC and TA in 0.5 mM melatonin have no significant difference from those in fruit treated with ddH_2_O (Table 1). Differences in ultrastructural changes were observed between the flesh of ddH_2_O and melatonin (0.5 mM) pears groups before and after storage. Furthermore, before the storage, cell wall structures were intact with a bright-dark-bright pattern, tightly arranged dark microfibrils, and darker-colored middle lamellae(Fig. 1A and B). After storage, the middle lamella were degraded in both the ddH_2_O and melatonin (0.5 mM) groups. Moreover, there was the loss of the bright-dark-bright pattern, microfibrils were loosely arranged, partial degradation, and lighter coloration. However, the degree of degradation of the cell wall in the melatonin (0.5 mM) group was reduced than that in the ddH_2_O group (Fig. 1C-F).

**Table 1:** Effects of treatment with melatonin on pear fruit firmness, SSC, TA and weight

Wild accession	Treatment	Firmness (N)	SSC (%)	Weight per fruit (g)	TA (%)
Zaoshu Shanli	0 d	11.20 ± 0.26c	10.50 ± 0.55a	52.26 ± 1.31a	1.62 ± 0.43a
	ddH_2_O	2.50 ± 0.26a	13.40 ± 0.72b	49.38 ± 1.22a	1.50 ± 0.53a
	0.5 mM	5.50 ± 0.30b	12.67 ± 0.67ab	50.13 ± 1.14a	1.56 ± 0.52a

a,b,c The means and standard deviations of three replicates are displayed. The same letter are not significantly different and different letters indicate a significant difference

This study observed that initially, the rates of ethylene production in both the ddH_2_O and exogenous melatonin treatment (0.5 mM) groups increased but then declined gradually during storage, increasing slowly from days 0—2, rapidly from days 2—6 and then gradually reached the peak on day 8 (ddH_2_O: 117.87 μL kg^−1^ h^−1^, melatonin: 97.25 μL kg^−1^ h^−1^), after which it gradually decreased through 10 d. Moreover, the rate of ethylene production in the melatonin (0.5 mM) group was always lower than that of the ddH_2_O group during storage, with reductions in the ethylene production levels by 78, 28, 21, 17, and 8% on days 2, 4, 6, 8, and 10, respectively (Fig. 1G). The respiration rate in the ddH_2_O group first decreased and then rapidly increased before again decreasing throughout the storage period, reaching a maximum of 42.52 mg kg^−1^ h^−1^ on 8 d. In the melatonin (0.5 mM) group, the respiration rate altered, with a maximum of 40.21 mg kg^−1^ h^−1^ on 4 d. The respiration rate in the melatonin group was reduced by 3, -5, 15, 10, and 5% on days 2, 4, 6, 8, and 10 of storage, respectively (Fig. 1H). Overall, relative to ddH_2_O treatment, the melatonin treatment suppressed the production and respiration rates in the pear fruit.

### KEGG pathway, GO annotation, and DEG enrichment analysis

**Fig. 2:**
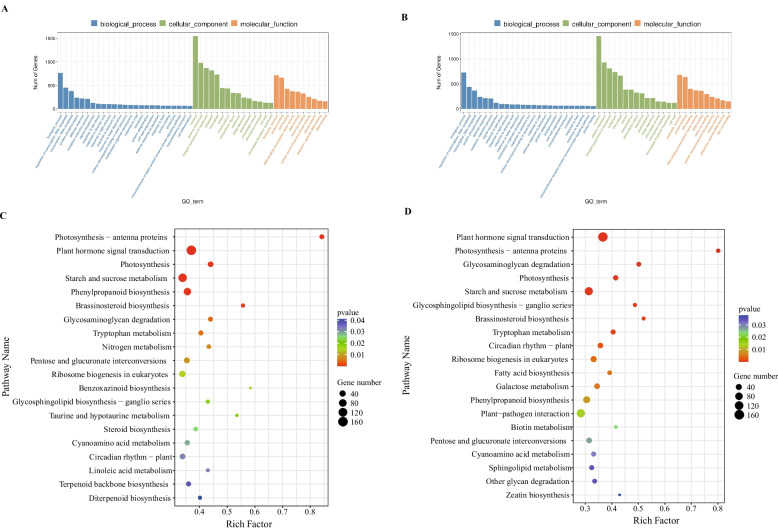
Analyses of DEmRNAs in ZSSL pears subjected to ddH_2_O and melatonin treatment. **A**, **C** GO (**A**) and KEGG (**C**) analyses for ddH_2_O vs. 0 d. **B**, **D** GO (**B**) and KEGG (**D**) analyses for the melatonin (0.5 mM) vs. 0 d

The mRNA and ncRNA analyses were conducted using transcriptomic data from pear flesh samples. The identified DEmRNA functions were assessed through GO and KEGG enrichment analyses. Comparable GO analyses were obtained for the ddH_2_O vs*.* 0 d and 0.5 mM vs*.* 0 d. The DEmRNAs enriched in biological processes included transcription regulation, DNA-templated transcription, and protein phosphorylation; those enriched in cellular components included the plasma membrane and nucleus, whereas the associated molecular functions included protein binding, ATP binding, and transcription activity in DNA binding (Fig. 2A and B). The comparisons of GO enrichment between the two groups indicated differences in cell wall-related pathways, as evident from differences in hydrolase activity, hydrolyzing O-glycosyl compounds, plant-type cell wall, and plasma membrane. Furthermore, the comparison between these groups' KEGG analyses revealed enrichment of the pathways for phenylpropanoid biosynthesis, starch and sucrose metabolism, and plant hormone signal transduction. This suggests that signal transduction was activated in pear cells during fruit storage, which ultimately affected the quality of fruit flesh (Fig. 2C and D).

### Changes in ncRNA expression patterns in response to exogenous melatonin treatment

**Fig. 3:**
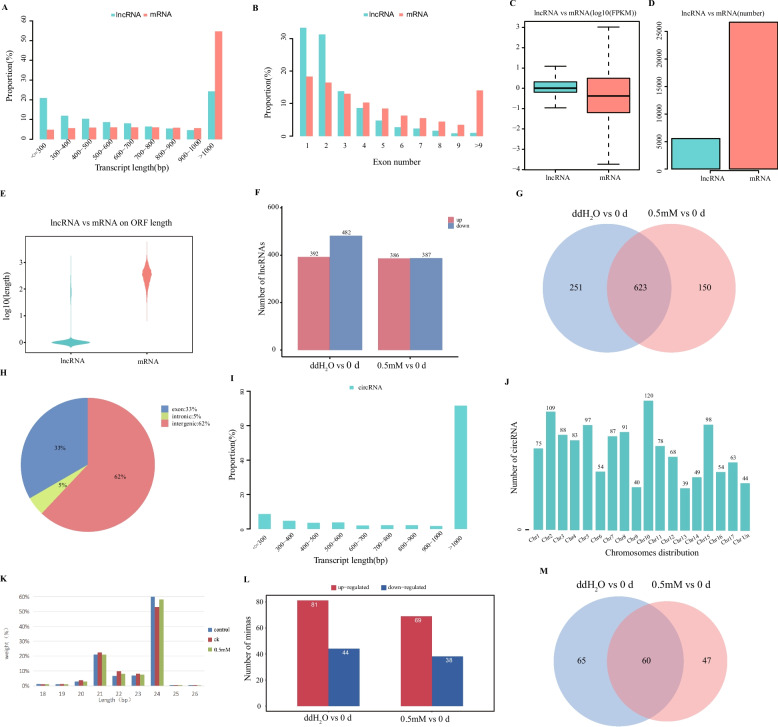
Analyses of lncRNA, circRNA, and miRNA expression in ZSSL pears subjected to ddH_2_O and exogenous melatonin treatments. **A**-**F**) lncRNAs and mRNAs analysis of transcript length (**A**), exon number (**B**), ORF length (**C**, **D**), FPKM values (**E**) and DElncRNA numbers (**F**). **G** Venn diagram of overlapping DElncRNAs between the ddH_2_O and exogenous melatonin (0.5 mM) treatment. **H**-**J** The identified circRNA’s distributions (**H**), sequence length (**I**), and chromosomal locations (**J**) analyses. **K** Small RNA sequence length distributions. **L** Numbers of DEmiRNAs. **M** Venn diagram of overlapping DEmiRNAs between the ddH_2_O and exogenous melatonin (0.5 mM) treatment

Relative to lncRNAs, mRNAs had a longer transcript length in this dataset, with approximately 86% of lncRNAs with < 1000 bp length and 80% comprising 1—2 exons (Fig. 3A and B). Furthermore, lncRNAs had a shorter open reading frame (ORF) and less varied FPKM values relative to mRNAs (Fig. 3C-E). Moreover, 874 DElncRNAs (392 upregulated, 482 downregulated) in ddH_2_O vs*.* 0 d and 773 DElncRNAs (386 upregulated, 387 downregulated) in melatonin (0.5 mM) vs*.* 0 d were identified (Fig. 3F). Of these, 623 DElncRNAs overlapped between the ddH_2_O vs*.* 0 d and melatonin (0.5 mM) vs*.* 0 d (Fig. 3G). In addition, 1349 circRNAs were detected across the analyzed samples, of which 62, 33, and 5% were intergenic, exonic, and intronic circRNAs, respectively (Fig. 3H). A majority of these circRNAs were either > 1000 bp or ≤ 300 bp long (Fig. 3I). The highest number of circRNAs was found on chromosome 10 (chr10), followed by chr2 and chr15 (Fig. 3J). A total of 1494 miRNAs were identified across all samples, the majority of which were 21—24 nucleotides long (Fig. 3K). Moreover, of all the identified miRNAs, 125 (81 upregulated, 44 downregulated) and 107 (69 upregulated, 38 downregulated) DEmiRNAs were identified in the ddH_2_O vs*.* 0 d and melatonin (0.5 mM) vs*.* 0 d, respectively (Fig. 3L), of which 60 overlapped between these two comparisons (Fig. 3M). Altogether, these data highlight the potential association between these different classes of ncRNAs and melatonin responses in pears.

### Changes in gene expression and ceRNA regulatory networks related to cell wall degradation after exogenous melatonin treatment

**Fig. 4:**
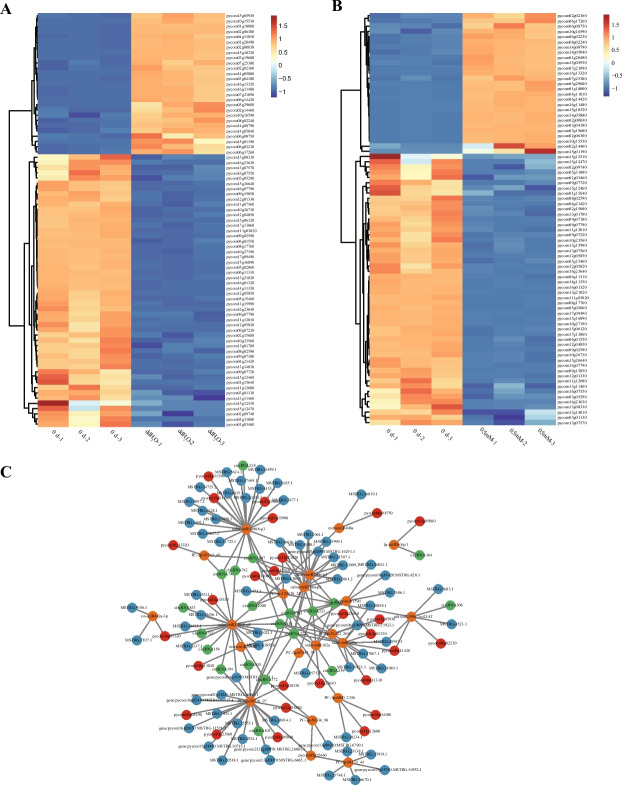
**A**, **B** Hierarchical clustering and heatmap analyses of DEGs associated with cell wall degradation for the ddH_2_O vs. 0 d (**A**) and melatonin (0.5 mM) vs. 0 d (**B**). **C** CeRNA networks related to cell wall degradation were established for ddH_2_O and melatonin-treated ZSSL pears, incorporating circRNAs, miRNAs, lncRNAs, and mRNAs. circRNA, green; lncRNA, blue; miRNA, yellow; mRNA, red

Consistent with the TEM findings, cell wall degradation was associated with the softening of fruit flesh after storage. Moreover, 80 genes associated with cell wall degradation were identified in transcriptomic analyses of ddH_2_O vs*.* 0 d and melatonin (0.5 mM) vs*.* 0 d (Fig. 4A and B). A total of 21 miRNA targets linked to cell wall degradation, 27 mRNAs, 65 lncRNAs, and 20 circRNAs were identified (Fig. 4C; Table S2). Mdm-miR10988-p3, mdm-miR11010-p5, and PC-3p-59718_97 were associated with more than 20 nodes in the established ceRNA network, suggesting that they could function as major regulatory components (Fig. 4C and Table S1).

**Fig. 5:**
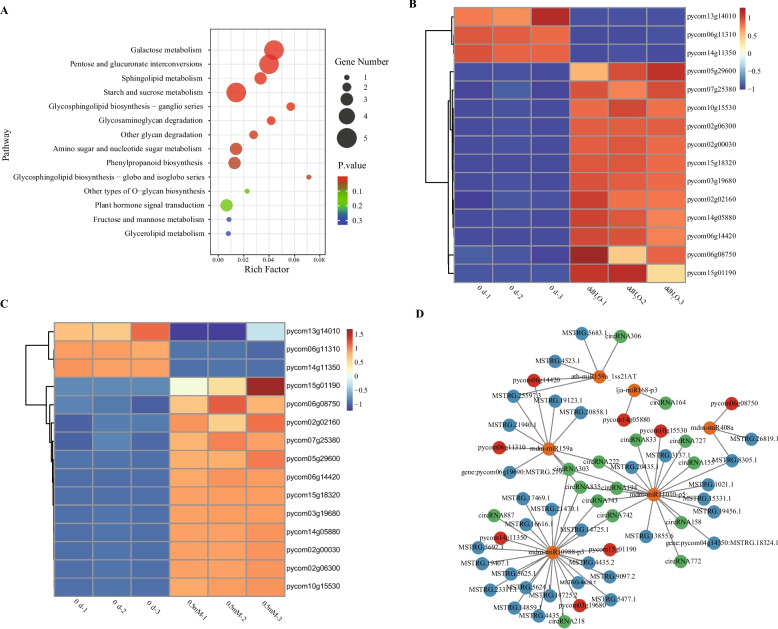
**A** KEGG enrichment analyses of genes associated with major cell wall degradation-related metabolic pathways. **B**, **C** Hierarchical clustering and heatmap analyses of DEGs associated with major cell wall degradation-associated metabolic pathways for the ddH_2_O vs*.* 0 d (**B**) and melatonin (0.5 mM) vs*.* 0 d (**C**) groups. **D** ceRNA networks related to major cell wall degradation-associated metabolic pathways were established for ddH_2_O- and exogenous melatonin-treated ZSSL pears, incorporating circRNAs (green), miRNAs (yellow), lncRNAs (blue), and mRNAs (red)

KEGG pathway analysis of the genes involved in cell wall disintegration indicated that these genes were major enriched in pathways associated with pentose and glucuronate interconversion, galactose metabolism, sphingolipid metabolism and starch and sucrose metabolism (Fig. 5A). Fifteen cell wall-related genes involved in these four primary pathways were analyzed (Fig. 5B and C; Table S2), showing that both *β-Gal* and *α-Gal* were observed to be up-regulated in the ddH_2_O and melatonin (0.5 mM) groups, with slight lower levels of up-regulation in melatonin (0.5 mM) group; expressions of pectate lyase and polygalacturonase were increased after storage in the ddH_2_O and melatonin (0.5 mM), however, the degree of up-regulation was slightly less pronounced in the melatonin treatment group; alterations in the expression of most starch and sucrose metabolism-related genes were also less pronounced in the melatonin (0.5 mM) relative to in ddH_2_O treatment. This potentially explaining observed pear cell wall degradation, which is consistent with the TEM findings. Eight mRNAs, 32 lncRNAs, and 15 circRNAs were found as putative targets of 6 miRNAs linked to important metabolic pathways (Fig. 5D; Table S3). Three miRNAs associated with major metabolic pathways (mdm-miR159a, mdm-miR11010-p5, and mdm-miR10988-p3) were also found to have common putative targets, suggesting that they may play the same biological functions (Fig. 5D; Table S3).

### Melatonin inhibits the synthesis of ethylene by downregulating the genes necessary for ethylene biosynthesis

**Fig. 6:**
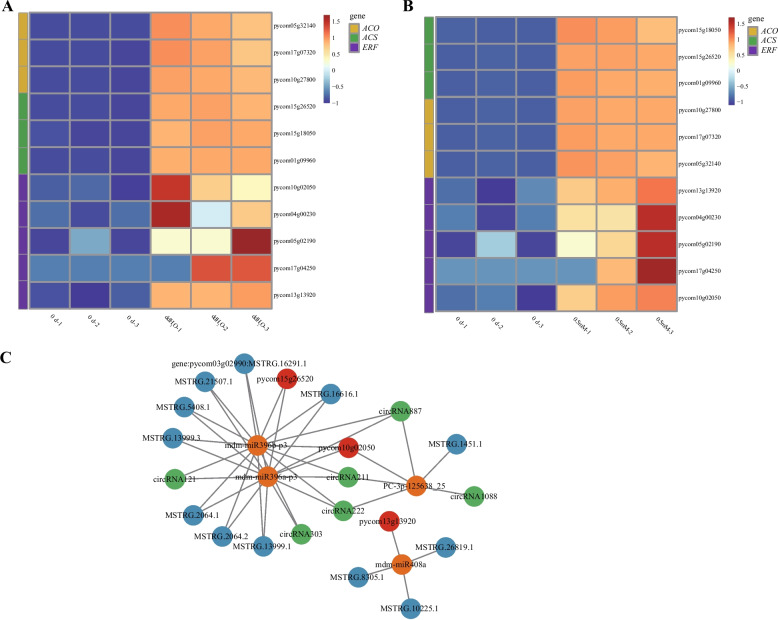
**A**, **B** Hierarchical clustering and heatmap analyses of DEGs associated with ethylene synthesis pathways in the ddH_2_O vs*.* 0 d (**A**) and melatonin (0.5 mM) vs*.* 0 d (**B**) groups. **C** ceRNA networks of ethylene synthesis pathways in ddH_2_O- and exogenous melatonin-treated ZSSL pears, incorporating circRNAs, miRNAs, lncRNAs, and mRNAs

Expression of most regulatory genes involved in the ethylene biosynthesis pathway, including *ACS* and *ACO* and five genes associated with ethylene-responsive transcription factors (*ERF*) (pycom17g04250, pycom10g02050, pycom13g13920, pycom05g02190, and pycom04g00230) were increased after storage in ddH_2_O treatment. Melatonin treatment was observed to slightly inhibit expression of most regulatory genes involved in the ethylene biosynthesis pathway, including *ACS* and *ACO* and also suppressed expression of five genes associated with ethylene-responsive transcription factors (*ERF*) in pear (Fig. 6A and B; Table S4). Here, four miRNAs implicated in ethylene metabolism were shown to target 3 mRNAs, 12 lncRNAs, and 6 circRNAs. In addition, 2 of these miRNAs were predicted to interact with shared targets (mdm-miR396b-p3 and mdm-miR396a-p3), indicating that their roles may overlap (Fig. 6C; Table S5).

### Regulatory changes related to aroma pathways and volatile organic compounds in response to exogenous melatonin treatment

**Fig. 7:**
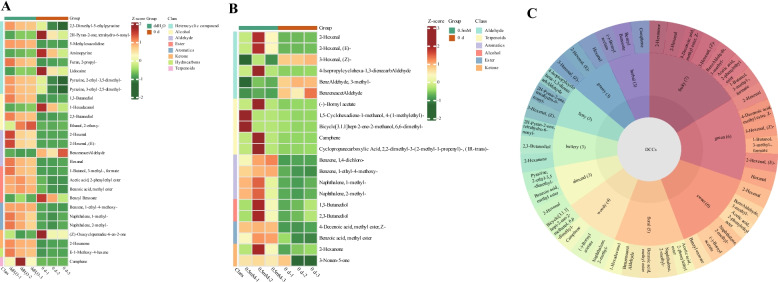
**A**, **B** Hierarchical clustering and heatmap analyses of aroma-related DCCs for the ddH_2_O vs*.* 0 d (**A**) and melatonin (0.5 mM) vs*.* 0 d (**B**) groups. **C** Flavor wheel analysis of all aroma-related DCCs in ZSSL pears under conditions of melatonin and ddH_2_O

**Fig. 8:**
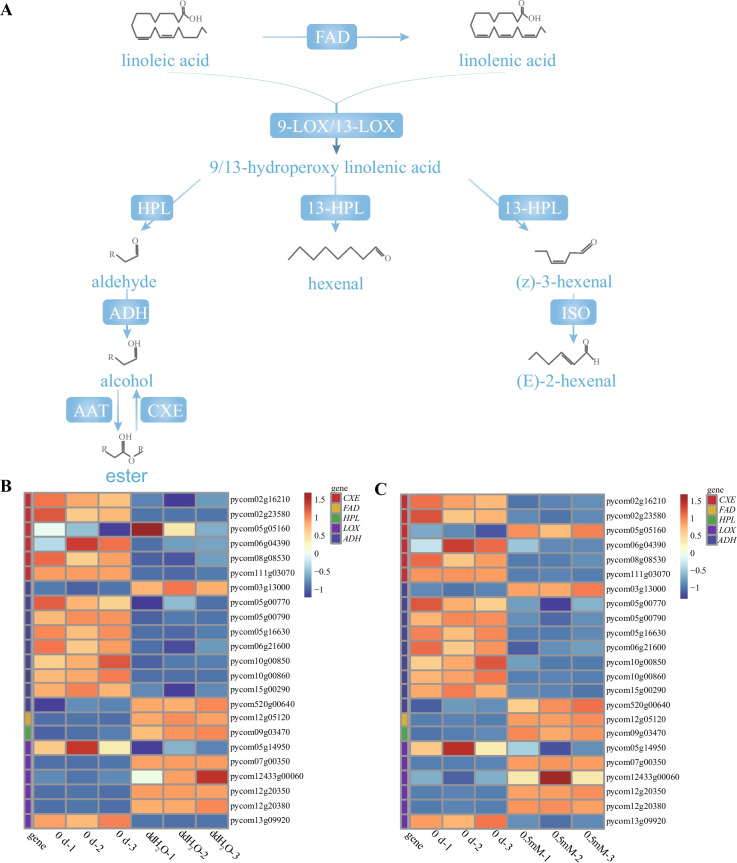
**A** DCC and DEG analyses of the LOX pathway in ZSSL pears treated with and without exogenous melatonin. **B**, **C** The number of genes found in target pathways associated with metabolism related to the aroma

**Fig. 9:**
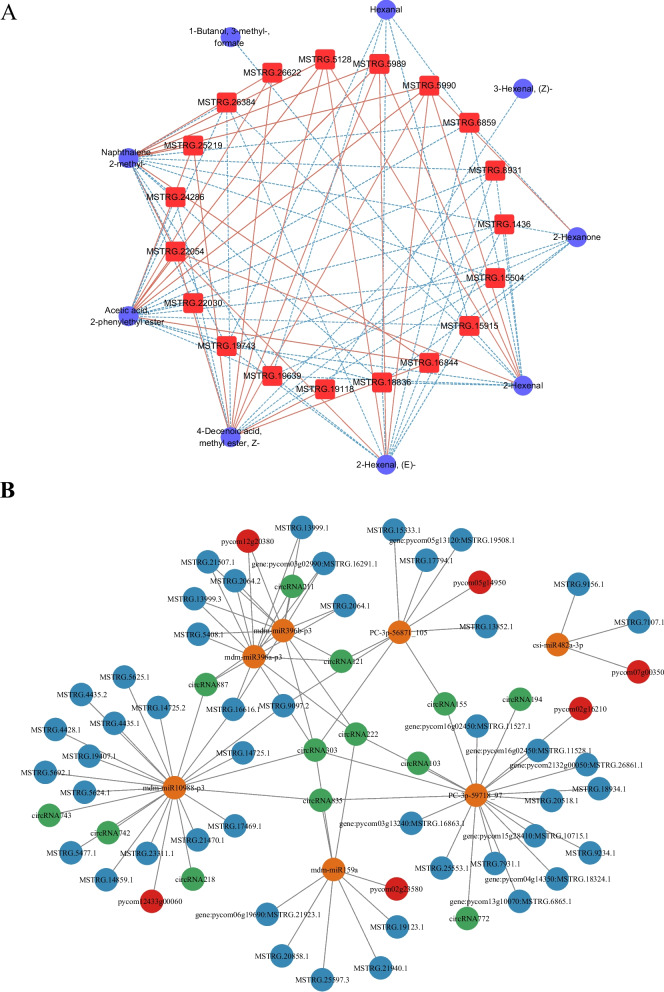
**A** Key genes related to the mechanistic basis for aroma production in ZSSL pears treated with melatonin as identified based on analyses of differentially abundant aromatic metabolites and mRNA regulatory networks. **B** ceRNA networks related to aroma were established for ddH_2_O and melatonin-treated ZSSL pears, incorporating circRNAs, miRNAs, lncRNAs, and mRNAs

To understand changes in aroma occurring in melatonin-treated pears after storage, an untargeted metabolomics investigation was conducted in the 0 d, ddH2O, and 0.5 mM samples. The metabolite compositions of these samples were analyzed (Fig. S1), with details provided in Table S6. The OPLS-DA score plots of the ddH2O group vs. the 0 d group and the 0.5 mM melatonin group vs. the 0 d group are shown in Fig. S2 and S3. Differentially changed compounds (DCCs) were identified using the criteria of VIP > 1 and absolute |Log2FC|≥ 1.0. Twenty-seven differentially changed compounds (DCCs; 20 upregulated and 7 downregulated) were identified in the pear fruit flesh after the melatonin (0.5 mM) vs*.* 0 d treatment. Whereas the ddH_2_O vs*.* 0 d treatment group indicated 21 DCCs (18 upregulated and 3 downregulated). Moreover, 9 DCCs were same in both the treatment groups (Fig. 7A and B; Table S7). All DCCs were associated with 3 aroma synthesis processes, including methylerythritol phosphate (MEP, such as camphene), shikimate (such as benzaldehyde, 3-methyl-), and lipoxygenase (LOX, such as 2-Hexenal, hexanal, and 1-Butanol, 3-methyl-, formate) pathways. Thus, exogenous melatonin treatment primarily altered the volatile aroma of pears by affecting these three pathways. Flavor wheel analysis identified the most critical flavors as fruity, green, and sweet (Fig. 7C), and the most annotated DCCs associated with these important flavors were involved in the LOX pathway. Furthermore, to better understand how mRNAs influence the synthesis of volatile aromatic compounds in pears, DEmRNAs were mapped to the LOX metabolite-related volatile aroma synthesis pathways (Fig. 8A). Twenty-three DEmRNAs were involved in LOX pathway, and expression of most of lipoxygenase, *FAD*, *ADH*, *HPL* and *CXE* genes of LOX pathway were slightly differentially expressed in ddH2O vs. 0 d and melatonin (0.5 mM) vs. 0 d groups. The different degrees of these alterations might explain the observed differences in volatile aromatic compound levels after the exogenous melatonin treatment (Fig. 8B and C; Table S8). Moreover, 9 DCCs were coregulated by 19 DEmRNAs by correlation analysis in LOX pathway (Fig. 9A; Table S9).

The LOX metabolic pathway was associated with 7 miRNAs, and a total of 6 mRNAs, 46 lncRNAs, and 13 circRNAs were identified as potential targets of these miRNAs. The ceRNA network indicated that mdm-miR10988-p3 and PC-3p-59718_97 were possible central regulators associated with more than 20 nodes. In addition, 6 of these LOX pathway-related miRNAs (mdm-miR396b-p3, mdm-miR396a-p3, mdm-miR10988-p3, mdm-miR159a, PC-3p-59718_97, and PC-3p-56871_105) were predicted to interact with overlapping targets, suggesting that they may exhibit similar functional roles (Fig. 9B; Table S10).

## Discussion

**Fig. 10:**
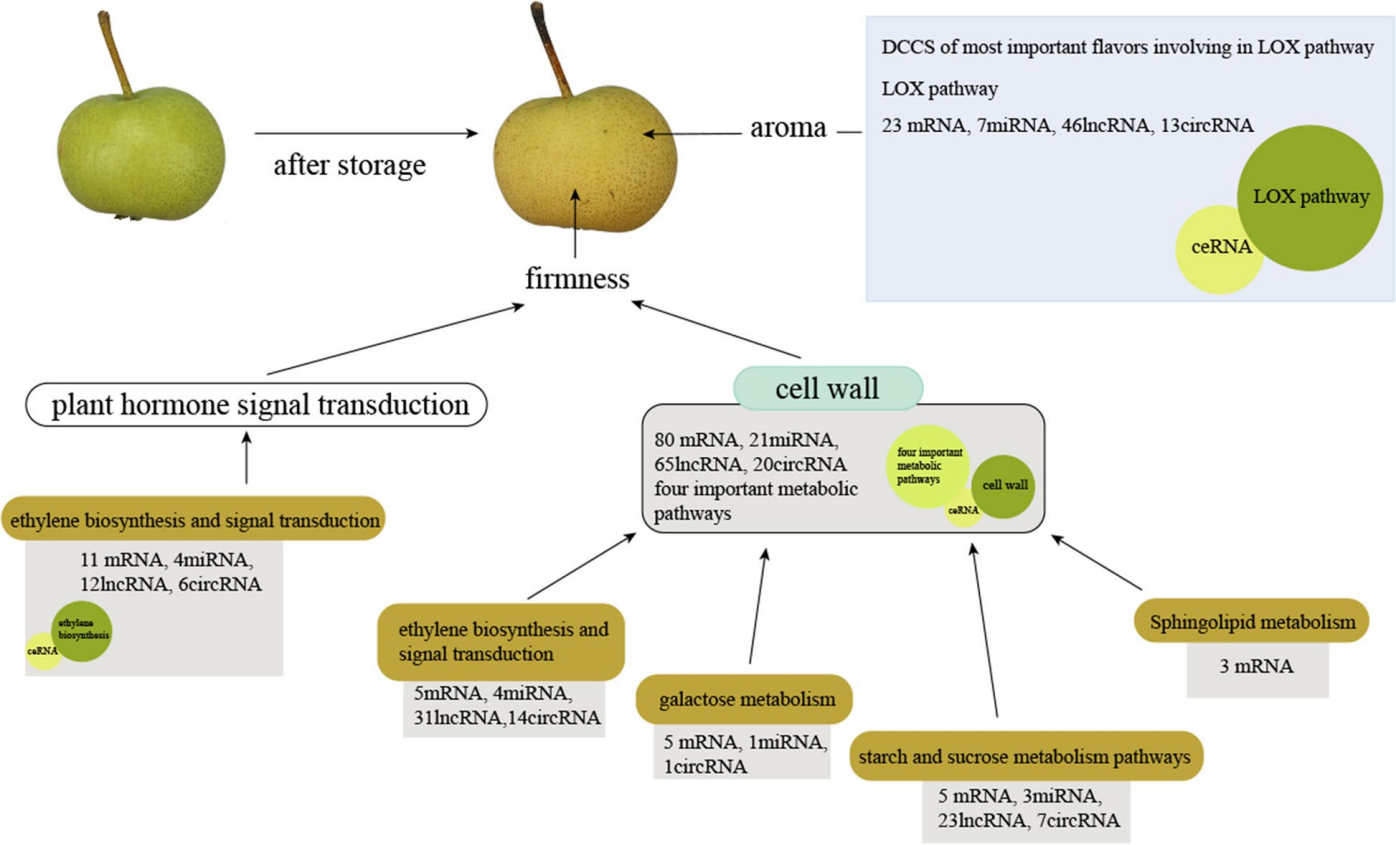
Schematic representation of postharvest pear fruits softening and aroma under ddH_2_O and melatonin treatment

This study undertook analysis of changes in ultrastructural features, volatile organic metabolites, mRNAs, and ncRNAs in the ZSSL pear with and without melatonin treatment, using TEM, metabolite profiling and whole-transcriptome RNA sequencing, to explain ZSSL fruit softening and changes in aroma after postharvest storage (Fig. 10).

Thickness of cell wall can affect fruit firmness [33]. The cell wall degradation initially occurs in the middle layer with the highest pectin content, and as the fruits continue to mature and age, the electron density of the middle layer decreases, pectin degrades, and larger intercellular spaces form [34]. In this study, TEM images showed that cell wall structures were intact, tightly arranged dark microfibrils, and darker-colored middle lamellae before storage. However, the cell wall structures, tightly arranged dark microfibrils, and middle lamella showed varying degrees of degradation in both ddH_2_O and exogenous melatonin treatment after storage, indicating degradation of cell wall caused pear fruit softening and exogenous melatonin treatment reduced rate of cell wall degradation. These results are consistent with the study of plum fruits after storage [35]. Ethylene is a key plant hormone that triggers climacteric fruit ripening [36]. In this study, ethylene production rate, expression of *ACO* and *ACS* genes related to ethylene biosynthesis and *ERF* genes were up-regulated in ddH_2_O after storage, while expression of *ACO* and *ACS* genes related to ethylene biosynthesis in melatonin treatment were slightly lower than that in ddH_2_O after storage and ethylene production rate and expression of *ERF* genes in melatonin treatment were lower than that in ddH_2_O after storage, suggesting that ethylene was involved in pear fruit softening and melatonin treatment could delay fruit softening by suppressing ethylene biosynthesis. This results are consistent with Hong et al.(2015) that ethylene promotes the fruits softening process [12] and Zhai et al. (2018) that melatonin delays the ripening of pears by repressing ethylene biosynthesis [20]. In this study, pentose and glucuronate interconversion, galactose metabolism, sphingolipid metabolism and starch and sucrose metabolism pathways were most important pathways that involved in pear fruit postharvest softening, and β-galactosidase (*β-Gal*), α-galactosidase (*α-Gal*), pectate lyase, polygalacturonase, endoglucanase and sucrose synthase were belonged to these most important pathways. β-galactosidase, α-galactosidase, pectate lyase, and polygalacturonase are integral to cell wall metabolism (Hu et al., 2024) [37]. In this study, expression of *β-Gal*, *α-Gal*, pectate lyase and polygalacturonase were observed to be up-regulated in the ddH_2_O treatment after storage, while melatonin delayed fruit softening by slightly inhibiting gene expression of *β-Gal*, *α-Gal*, *PG* and pectate lyase, which is consistent with the TEM findings. Sun et al. (2022) revealed that exogenous melatonin treatment was sufficient to inhibit polygalacturonase and β-galactosidase expression and jujube fruit softening [38]. Paniagua et al. (2016) used β-galactosidase silenced cell lines and revealed greater firmness than that for wild-type strawberry fruit [39] and α-galactosidase was involved in the cell wall polysaccharide metabolic pathway [37]. We speculate that difference were slightly for the following reasons: Material variability, the material in this study was wild *Pyrus ussuriensis* Maxim. resource which is known to have strong resistance and self-protection functions, suggesting that it may have been less sensitive to exogenous melatonin. In previous studies, melatonin treatment was applied to three different cultivars of *Pyrus communis* L., resulting in differences in the expression degrees of fruit softening-related genes such as *ACS* and *PG* among three different cultivars [20, 21]. This study also used fruit flesh tissue, which are shielded by the peel from direct contact with melatonin.

The aroma can impact the sensory qualities of fruit, thereby influencing consumer satisfaction. Concentrations of (E)-2-hexenal and hexanal decreased during peach postharvest softening [40], while that increased in ZSSL fruit under ddH_2_O treatment during postharvest softening in this study. Hydroperoxide lyase (HPL) can metabolized hydroperoxides into various C6 and C9 aldehydes [41]. Changes in aroma under ddH_2_O treatment were mainly in LOX pathway and aldehydes are important fruit aroma components, 2-Hexenal and 2-Hexenal, (E)- were up-regulated expressed in ZSSL pear under ddH_2_O treatment, might due to changes in expression levels of HPL, which led to changes in aldehyde compounds, which was consistent with Yue et al. (2022), who showed that *ZjHPL* expression and increased (E)-2-hexenal levels in *jujube* [42]. 2-Hexenal, 2-Hexenal, (E)- and 3-Hexenal, (Z)- were differentially expressed in ZSSL pear under exogenous melatonin treatment, might due to slightly changes in expression levels of HPL, which led to changes in aldehyde compounds,and result were supported by Liu et al. (2019), who showed that melatonin inhibited *PbHPL* expression and reduced hexanal and (E)-hex-2-enal levels in Korla [43]. We speculate that reasons for slightly difference in most of aroma-related genes expression were same as the slight differences observed in fruit softening.

Similar competing endogenous RNA (ceRNA) networks have been identified as essential regulators of many physiological and pathological processes, such as miRNAs can suppress complementary target mRNAs translation, while ceRNAs can competitively bind these miRNAs to abrogate their function. Classes of ceRNAs include certain mRNAs, lncRNAs and circRNAs [44]. In our study, number of lncRNAs was large more than number of circRNAs in ceRNA, which showed that lncRNAs was another important component of ceRNA, and similar results in maize development [45]. MiRNAs, as most important component of ceRNA, played an important role in fruit softening and aroma synthesis. Targets of miR159a were lipoxygenase related to LOX pathway in pear [46] and *ACO* related to ethylene biosynthesisin in sweet cherry [47]. In this study, *CXE* in LOX pathway, pectate lyase in pentose and glucuronate interconversion pathway and glucan endo-1,3-beta-glucosidase in starch and sucrose metabolism pathway were targeted by mdm-miR159a. In contrast to previous studies, miR159a can affect fruit softening and aroma formation, but the target genes were not different. This result might be caused by different species and different types of pear resources. For the moment, there was few information about function of miR408a, and previous studies had shown that miR408a was mainly involved in response to stress resistance in soybean [48] and birch [49]. While ethylene-responsive transcription factors and endoglucanase related to starch and sucrose metabolism pathway were targeted by mdm-miR408a, that showed mdm-miR408a could be invoved in fruit softening in this study. Targets of cpa-miR396 were *ACS* related to ethylene biosynthesisin and ethylene-responsive transcription factors in papaya [50] that was same as this study, while 9 s-lipoxygenase related to LOX pathway was also target gene for mdm-miR396a/b-p3 in pear in this study. In network of ZSSL pear fruit, 16 lncRNAs and 4 mRNAs act as miRNA sponges by targeting mdm-miR10988-p3 to regulate cell wall and aroma biosynthetic process, which indicated functional diversity of mdm-miR10988-p3 and importance of mdm-miR10988-p3 in the ceRNA regulatory network.

## Conclusion

In summary, ZSSL pear fruit softening was caused by four important pathways associated with cell wall degradation, promoted synthesis of pear fruit aroma components, while exogenous melatonin treatment can slow down ZSSL fruit softening and alter aroma in pears. This study also observed that with and without melatonin treatment affected fruit aroma major through the LOX pathway, modulating production of metabolites. The ceRNA analysis indicated that mdm-miR396b-p3 and mdm-miR396a-p3 in the ethylene metabolic pathway may have the same functions. Mdm-miR10988-p3, mdm-miR11010-p5, and PC-3p-59718_97 were identified as the core regulators of cell wall degradation, while mdm-miR159a, mdm-miR11010-p5, and mdm-miR10988-p3 might play comparable functional roles in cell wall degradation. Moreover, mdm-miR10988-p3 and PC-3p-59718_97 were core regulators of the LOX pathway, whereas mdm-miR396b-p3, mdm-miR396a-p3, mdm-miR10988-p3, mdm-miR159a, PC-3p-59718_97, and PC-3p-56871_105 related to LOX metabolic pathway might have similar functions.

## Supplementary Information

Supplementary Material 1.

## Data Availability

Data presented in this manuscript are included in supplemental tables and raw data was submitted to NCBI under the BioProject ID: PRJNA1227437.

## Abbreviations

SSC: Soluble solid content
CW: Cell wall
PM: Plasmolemma
CJ: Cell junction
FAD: Fatty acid desaturase
HPL: Hydroperoxide lyase
CXE: Carboxyl esterase
DCCs: Differentially changed compounds
β-Gal: β-Galactosidase
PG: Polygalacturonase
ACS: 1-Aminocyclopropane-1-carboxylate synthase
ACO: 1-Aminocyclopropane-1-carboxylate oxidase
TA: Titratable acidity
ML: Middle lamella
SG: Starch grain
MF: Microfibril
LOX: Lipoxygenase
ADH: Alcohol dehydrogenase
TEM: Transmission electron microscopy
DEGs: Diferentially expressed genes
α-Gal: α-Galactosidase
PL: Pectate lyase
